# Combining EGFR and KRAS G12C Inhibitors for KRAS G12C Mutated Advanced Colorectal Cancer

**DOI:** 10.33696/cancerimmunol.6.086

**Published:** 2024

**Authors:** Hirotaka Miyashita, David S. Hong

**Affiliations:** 1 Dartmouth Cancer Center, Lebanon, NH, United States; 2 The University of Texas MD Anderson Cancer Center, Houston, TX, United States

**Keywords:** Clinical trials, Colorectal cancer, KRAS, KRAS G12C inhibitors, RTK inhibitors

## Abstract

KRAS is a commonly mutated gene in advanced colorectal cancer (CRC). Recently, inhibitors of KRAS G12C were developed and have shown promising efficacy for KRAS G12C mutated non-small cell lung cancer. However, KRAS G12C inhibitor monotherapy has not demonstrated excellent efficacy for KRAS G12C mutated advanced CRC due to multiple resistance mechanisms, especially receptor tyrosine kinase (RTK) signaling activation. To overcome this resistance mechanism, various combinations of epithelial growth factor receptor (EGFR) and KRAS G12C inhibitors, including panitumumab plus sotorasib, have been investigated in clinical trials.

The combination of EGFR and KRAS G12C inhibitors for KRAS G12C mutated CRC demonstrated overall response rates ranging from 26% to 62.5% in seven clinical trials of phase I to III, whose data are available so far. The median progression-free survival in these trials ranged from 3.9 to 8.1 months. These efficacy data suggest that KRAS G12C inhibitor combination with EGFR inhibitors is more effective for KRAS G12C mutated advanced CRC than KRAS G12C inhibitor monotherapy. They also showed reasonable safety of the combination regimen. Based on these results, phase III clinical trials are being conducted to investigate EGFR and KRAS G12C inhibitor combinations as a first or second-line treatment for KRAS G12C mutated advanced CRC. Furthermore, other KRAS G12C inhibitors, KRAS G12D inhibitors, and pan-RAS inhibitors are being developed, which could make more patients with advanced CRC eligible for KRAS inhibition.

## Graphical Abstract

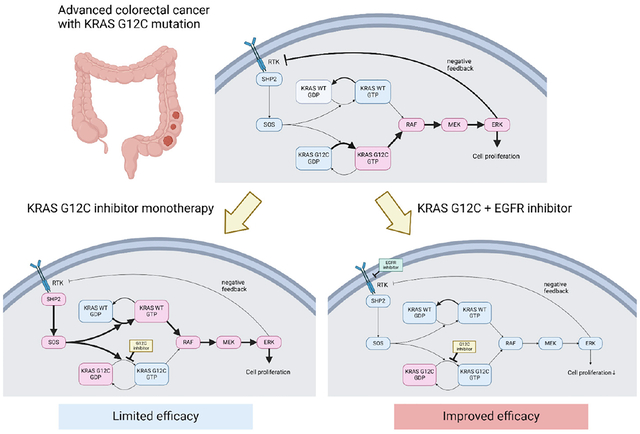

## Introduction

Colorectal cancer (CRC) is the third most common cause of cancer mortality in the US, and 53,010 deaths from CRC are estimated in 2024 [1]. The 5-year survival for CRC with distant metastasis is only 14%, [1] for which new treatments are investigated.

Mutations of Kristen rat sarcoma viral oncogene homologue (KRAS) are commonly detected in advanced CRC in up to 40–50% of all cases [2,3]. KRAS mutated advanced CRC has a poorer prognosis than KRAS wild-type CRC [4]. In advanced KRAS mutated CRC cases, KRAS G12D mutation is the most common, followed by G13D, G12V, G12S, and G12C mutations.

Because of the high prevalence of KRAS mutation across many types of cancer [5], KRAS inhibitors have been investigated vigorously. Still, KRAS inhibition had been unsuccessful until recently due to its inaccessible binding surface and high affinity to guanosine triphosphate [6]. However, sotorasib, a small molecule binding to a specific KRAS G12C mutation, was developed and showed promising efficacy for KRAS G12C mutated NSCLC [7], leading to the approval by the Food and Drug Administration (FDA) in May 2021. This has proven that KRAS is a “druggable” target for cancer treatment, and many other KRAS G12C inhibitors and inhibitors of other KRAS mutations are currently under investigation.

## Limited Efficacy of KRAS G12C Inhibitor Monotherapy for Advanced CRC

As of April 2024, two KRAS G12C inhibitors have been approved by the FDA, including sotorasib and adagrasib, as a subsequent line of treatment for KRAS G12C mutated non-small cell lung cancer (NSCLC). In CodeBreak 100, a phase II trial of sotorasib monotherapy for patients with KRAS G12C mutated advanced NSCLC, previously treated with standard therapies, an objective response rate (ORR) of 37.1% with median progression-free survival (mPFS) of 6.8 months were observed. KRYSTAL-1 is a phase I/II trial of adagrasib monotherapy for previously treated advanced NSCLC with KRAS G12C mutation, and it demonstrated an ORR of 42.9% and mPFS of 6.5 months. Based on these data, sotorasib and adagrasib were granted accelerated approval from the FDA in May 2021 and December 2022, respectively.

In contrast to the promising efficacy of KRAS G12C inhibitor monotherapy for KRAS G12C mutated advanced NSCLC, previous clinical trials failed to show clear benefit of KRAS G12C inhibitor monotherapy for KRAS G12C mutated CRC (Table 1). In the phase I part of CodeBreaK 100, 42 patients with advanced KRAS G12C mutated CRC were treated with sotorasib monotherapy, and ORR was 7.1% (3 patients) and mPFS was 4.0 months [8]. In the phase II part of CodeBreaK 100, sotorasib monotherapy was given to 62 patients with KRAS G12C mutated advanced CRC. It demonstrated ORR of 9.2% (6 patients) with mPFS of 4.0 months [9]. Adagrasib monotherapy for pretreated KRAS G12C mutated advanced CRC was evaluated in KRYSTAL-1, where 44 patients with CRC received adagrasib monotherapy, and an ORR of 19% with mPFS of 5.6 months was reported [10]. Divarasib is another covalent KRAS G12C inhibitor given to 55 patients with previously treated KRAS G12C mutated advanced CRC in a phase I trial [11]. This trial demonstrated ORR of 29.1% with mPFS of 5.6 months, while the NSCLC sub-cohort of the same trial showed 53.4% of ORR and 13.1 months of mPFS.

The suboptimal response to targeted therapy as monotherapy was observed when BRAF inhibitors were investigated for BRAF V600E mutated CRC [12]. Prahallad *et al*. demonstrated that BRAF inhibition leads to rapid feedback activation of epithelial growth factor receptor (EGFR), allowing continued tumor proliferation even with BRAF inhibition. They also revealed *in vivo* and *in vitro* that the blockade of EGFR with BRAF has a strong synergistic effect on BRAF mutated CRC [13]. This concept was proven clinically in a phase Ib trial combining vermurafenib (a BRAF inhibitor) with cetuximab (an EGFR inhibitor) and irinotecan for BRAF V600E mutated CRC [14]. Followed by a phase II trial to compare irinotecan plus cetuximab with or without vemurafenib [15]. Eventually, the combination of encorafenib (a BRAF V600E inhibitor) and cetuximab has shown survival benefits in BRAF V600E mutated advanced CRC, leading to the first FDA approval.

**Figure 1.**
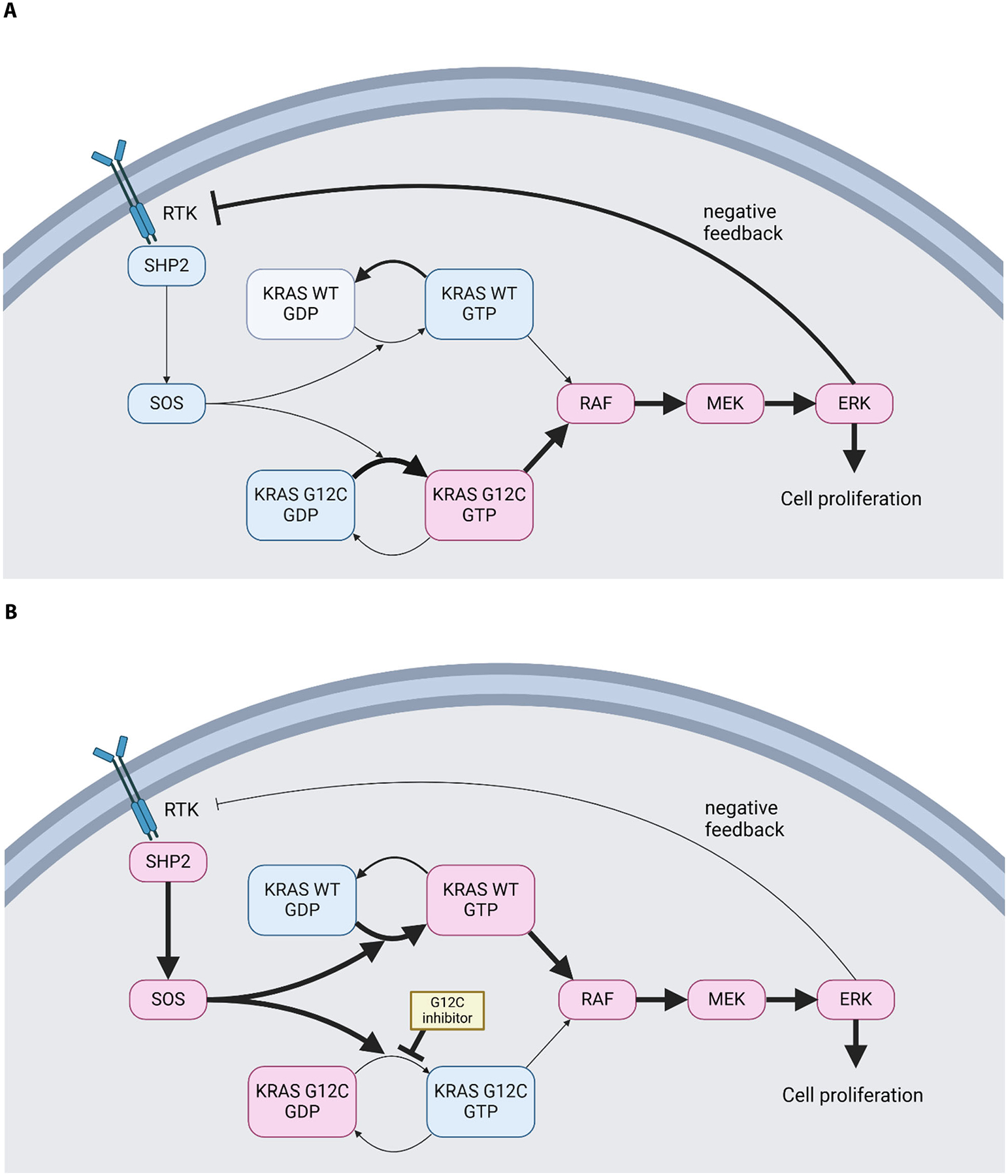

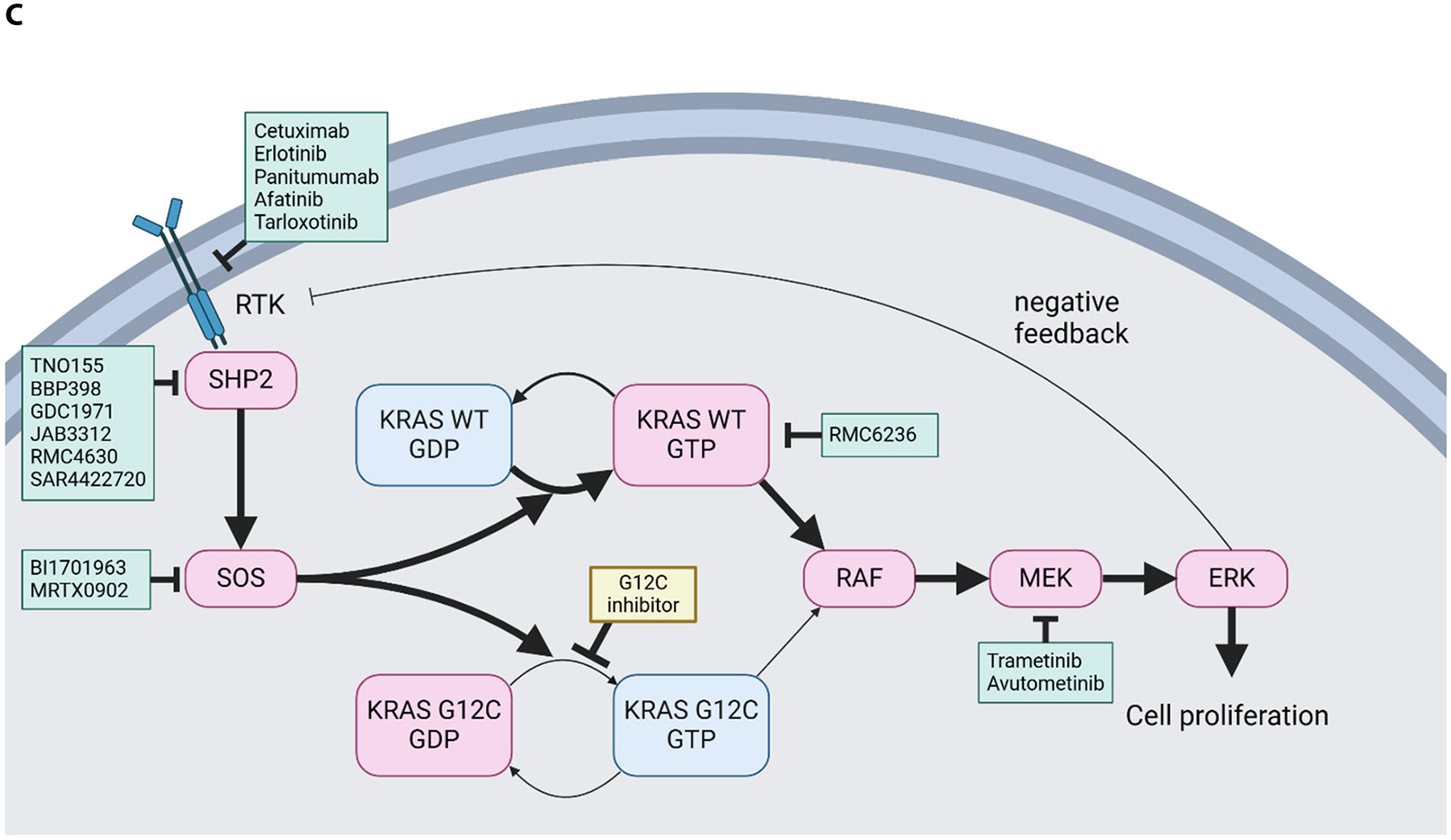
Negative feedback of MAPK signaling in KRAS G12C mutated colorectal cancer. **A**. In KRAS G12C mutated CRC, MAPK signaling is persistently activated, leading to the negative feedback to RTK, suppressing the activation of wild-type KRAS. **B**. By inhibiting KRAS G12C, MAPK signaling is initially suppressed, but due to suppressed negative feedback to RTK by ERK, RTK and downstream cascade are activated, including wild-type KRAS. This leads to the rebound activation of the MAPK pathway. **C**. In green boxes, rational interventions to combine with KRAS G12C inhibitors and their target to suppress ERK rebound.

In KRAS G12C inhibition for CRC, multiple resistance mechanisms have been revealed, including collateral signaling and primary and acquired genomic co-alterations [16]. Among them, receptor tyrosine kinase (RTK), including EGFR, feedback activation is one of the most critical mechanisms of primary resistance to KRAS G12C inhibitor monotherapy for CRC, as was seen in BRAF inhibitor monotherapy. *In vitro* study demonstrated that KRAS G12C mutated CRC cell lines have high basal RTK activity, and inhibiting KRAS G12C induces increased phospho-ERK rebound (Figures 1A and 1B). Based on these findings, the combination of EGFR and KRAS G12C inhibitors was evaluated *in vivo*, showing a high efficacy in patient-derived xenografts [17]. This preclinical evidence has prompted multiple early-phase clinical trials to assess the combination of EGFR and KRAS G12C inhibitors for KRAS G12C mutated advanced CRC.

## Current Evidence on Combining RTK and KRAS G12C Inhibitors for KRAS G12C Mutated CRC

Panitumumab and cetuximab are the EGFR inhibitors combined with KRAS G12C inhibitors in clinical trials, the results of which are available so far (Table 2). In a dose-expansion cohort of CodeBreaK 101 sub-cohort, 40 patients with chemotherapy-refractory KRAS G12C mutated metastatic CRC were given panitumumab 6 mg once every two weeks with sotorasib 960 mg daily. Twelve patients (30%) showed confirmed objective response; the mPFS was 5.7 months. Treatment-related adverse events with grade 3 or higher happened in 27% of patients with dermatologic events most common, which suggests an acceptable safety profile of the regimen [18]. The same regimen was investigated in a phase III trial (CodeBreaK 300) for 53 patients with chemotherapy-refractory KRAS G12C mutated advanced CRC without previous KRAS G12C inhibition [19]. They compared this regimen with the same combination at a lower sotorasib dose (240 mg daily, 53 patients) and standard care chemotherapy regimens (trifluridine–tipiracil or regorafenib). In the sotorasib 960 mg cohort, ORR was 26.4%, and mPFS was 5.6 months, while in the sotorasib 240 mg cohort, they were 5.7% and 3.9 months, respectively. In the chemotherapy arm, ORR and mPFS were 0% and 2.2 months, respectively. Sotorasib 960 mg with panitumumab showed a hazard ratio for PFS of 0.49 (95% confidence interval, 0.30 to 0.80) compared to standard care chemotherapy. The regimen of sotorasib 960 mg daily with panitumumab 6 mg every other week was further combined with standard-dose FOLFIRI every two weeks in a subprotocol of CodeBreaK 101 [20]. Forty-six patients with previously treated KRAS G12C mutated metastatic CRC demonstrated ORR of 55% with a reasonable safety profile.

On the other hand, cetuximab was combined with adagrasib in KRYSTAL-1, a phase I/II trial. Ninty-four patients with pretreated KRAS G12C mutated metastatic CRC were given adagrasib 600 mg twice daily and cetuximab with an initial loading dose of 400 mg per square meter of body-surface area, followed by 250 mg per square meter every week or 500 mg per square meter every other week. The ORR was 34.0%, and mPFS and median OS were 6.9 and 15.9 months, respectively. 27.7% of patients had grade 3–4 TRAEs, with nausea being the most common [21]. Weekly cetuximab (400 mg per square meter followed by 250 mg per square meter) was combined with divarasib in a phase Ib trial [22]. Among 24 patients with no previous KRAS G12C inhibition, 23 received divarasib 400 mg daily, and one received divarasib 200 mg daily with weekly cetuximab. They showed ORR of 62.5% with mPFS of 8.1 months. Five patients who had received KRAS G12C inhibition previously received divarasib 200 mg daily (2 patients) or 400 mg daily (3 patients) and showed ORR of 60%. Overall, acceptable toxicity was reported. In a phase II clinical trial, a standard dose of cetuximab was combined with D-1553, another KRAS G12C inhibitor. Twenty-nine patients with pretreated KRAS G12C mutated metastatic CRC without previous KRAS G12C inhibition were given D-1553 600 mg two times daily with cetuximab. The preliminary response rate was 45%, and mPFS was 7.6 months. Grade 3–4 TRAEs were observed in 12.5% [23]. Moreover, a preliminary report of the phase I trial to combine standard dose cetuximab with another oral, potent, highly selective KRAS G12C inhibitor, LY3537982, was recently reported [24]. In this phase I study, 49 patients with previously treated advanced KRAS G12C mutated CRC, without prior KRAS G12C inhibition, were given LY3537982 100 mg or 150 mg twice daily with cetuximab. The ORR was 45%, and the mPFS was 7.6 months. Most common TRAEs were dermatologic events, and no TRAE leading to LY3537982 discontinuation was observed.

## Summary and Future Direction

In contrast to the limited efficacy of KRAS G12C inhibitor monotherapy for KRAS G12C mutated advanced CRC, the combination of RTK and KRAS G12C inhibitors has shown promising efficacy with reasonable safety. Although the FDA has not granted full approval to these combination regimens, they could be a part of treatment for advanced KRAS G12C mutated CRC soon when the evidence matures. However, many questions have yet to be answered: Which RTK and KRAS G12C inhibitor combination has the best efficacy and safety? Which line of treatment should this combination be considered? Should chemotherapy be added to the combination of RTK and KRAS G12C inhibitors? Numerous clinical trials try to answer these questions (Table 3). For example, CodeBreaK 301 (NCT06252649) is a phase III trial of panitumumab plus sotorasib for treatment naïve metastatic KRAS G12C mutated CRC cases. KRYSTAL-10 (NCT04793958) is a phase III trial of adagrasib combined with cetuximab as a second-line treatment for KRAS G12C mutated advanced CRC. On the other hand, adagrasib is a strong CYP3A4 inhibitor and requires special dosage consideration to combine with irinotecan, for which a clinical trial is also ongoing (NCT05722327).

In addition to RTK, there are other possible targets to suppress bypass signaling of KRAS G12C inhibition, such as SHP2, SOS, wild-type KRAS, and MEK (Figure 1C). These molecules play critical roles in the RTK-RAS-MAPK pathway, and co-inhibiting these molecules with KRAS G12C has revealed efficacy in preclinical models [25,26]. Multiple clinical trials of these combinations are ongoing, which may reveal other reasonable combinations for KRAS G12C mutated CRC.

Lastly, KRAS inhibition goes beyond KRAS G12C, including KRAS G12D and pan-RAS inhibition. MRTX1133 is a KRAS G12D inhibitor, showing antitumor efficacy in-vivo and in-vitro [27], and is currently in a phase I/II clinical trial. (NCT05737706) Another new KRAS inhibitor, protein degrader, which degrades mutated KRAS through ubiquitination, is also being developed. ASP3082 is a KRAS G12D degrader and has demonstrated antitumor efficacy in PDAC, CRC, and NSCLC preclinical models [23]. A phase I clinical trial of ASP3082 is ongoing. (NCT05382559) Beyond KRAS G12C or G12D, pan-RAS inhibitors, such as RMC6236 and RMC7977, are being developed [28–30]. Since KRAS G12C mutation is relatively rare in advanced CRC, developing therapeutics to inhibit a wider variety of KRAS inhibition is needed to improve further the treatment for KRAS mutated CRC.

## Figures and Tables

**Table 1.** Data of KRAS G12C inhibitor monotherapy for KRAS G12C mutated advanced colorectal cancer.

Medication (NCT number)	Phase	Population	N	Efficacy	Reference
Sotorasib (NCT03600883)	1	Previously treated KRAS G12C mutated CRC	42	RR: 7.1% mPFS: 4.0 m	[8]
Sotorasib (NCT03600883)	2	Previously treated KRAS G12C mutated CRC	62	RR: 9.2% mPFS: 4.0 m	[9]
Adagrasib (NCT03785249)	2	Previously treated KRAS G12C mutated CRC	44	RR: 19% mPFS: 5.6 m	[10]
Divarasib (NCT04449874)	1	Previously treated KRAS G12C mutated CRC	55	RR: 29.1% mPFS: 5.6 m	[11]

CRC: Colorectal Cancer; N: Number of Participants; RR: Response Rate; mPFS: Median Progression-Free Survival

**Table 2.** Data of KRAS G12C inhibitor combined with RTK inhibitor for KRAS G12C mutated advanced colorectal cancer.

Combination and NCT number	Phase	Population	N	Toxicity data	Efficacy data	Reference
Panitumumab + Sotorasib NCT04185883 (CodeBreaK 101)	1b	Previously treated metastatic CRC	48*	G3–4 TRAE: 27% (most commonly dermatologic) TRAE to d/c regimen: 0%	In the dose expansion cohort (N = 40) RR: 30% mPFS:5.7 m mOS: 15.2 m	[18]
Panitumumab + Sotorasib NCT05198934 (CodeBreaK 300)	3	Previously treated metastatic CRC	106	960 mg sotorasib cohort (N=53) Common TRAE: hypomagnesemia, rash, dermatitis acneiform G3–4 TRAE: 36% TRAE to d/c regimen: 4% 240 mg sotorasib cohort (N=53) Common TRAE: hypomagnesemia, rash, dermatitis acneiform G3–4 TRAE: 30% TRAE to d/c regimen: 2%	960 mg sotorasib cohort (N=53) RR: 26.4% mPFS: 5.6 m 240 mg sotorasib cohort (N=53) RR: 5.7% mPFS: 3.9 m	[19]
Panitumumab + FOLFIRI + Sotorasib NCT04185883 (CodeBreaK 101)	1b	Previously treated metastatic CRC	46	Common TRAE: dermatitis acneiform, dry skin, nausea and stomatitis G3–4 TRAE: 43% (most commonly dermatologic) TRAE to d/c regimen: 2% for sotorasib (ALT increased), 4% for panitumumab and 24% for FOLFIRI	RR: 55%	[20]
Cetuximab + Adagrasib NCT03785249 (KRYSTAL-1)	1/2	Previously treated metastatic CRC	94	Common TRAE: nausea, vomiting, diarrhea G3–4 TRAE: 27.7% TRAE to d/c regimen: 0% for adagrasib, 8.5% for cetuximab	RR: 34.0% mPFS: 6.9 m mOS: 15.9 m	[21]
Cetuximab + Divarasib NCT04449874	1b	Advanced or metastatic CRC (does not specify prior KRAS inhibition)	29	Common TRAE: rash, diarrhea, nausea G3–4 TRAE: 45% TRAE to d/c regimen: 0% for divarasib, 3.4% for cetuximab (rash)	Previous G12C inhibition (N=5) RR: 60% G12C inhibition naïve (N=24) RR: 62.5% mPFS: 8.1 m	[22]
Cetuximab + D1553 NCT04585035	2	Metastatic CRC with no prior KRAS G12C treatment	40	Common TRAE: rash, increased AST/ALT, paronychia G3–4 TRAE: 12.5% TRAE to d/c regimen: 2.5% (cetuximab related)	RR: 45.0% (not all confirmed) mPFS: 7.6 m	[23]
Cetuximab + LY3537982 NCT04956640	1	Pretreated advanced CRC with no prior KRAS G12C treatment	49	Common TRAE: dermatitis acneiform, dry skin, diarrhea, hypomagnesemia G3–4 TRAE: 20% TRAE to d/c regimen: 2% for cetuximab but none for LY3537982	RR: 45% mPFS: 7.6 m	[24]

AST: Aspartate Transferase; ALT: Alanine Aminotransferase; CRC: Colorectal Cancer; N: Number of Participants; mOS: Median Overall Survival; mPFS: Median Progression-Free Survival; TRAE: Treatment-Related Adverse Event

* Eight patients were in a dose-exploration cohort, and 40 patients were in a dose-expansion cohort

**Table 3.** Ongoing clinical trials of RTK plus KRAS G12C inhibitor for KRAS G12C mutated colorectal cancer.

Combination	NCT number	Phase	Population	Current status*
Panitumumab + Sotorasib (CodeBreaK 301)	NCT06252649	3	Treatment-naïve metastatic CRC	Not yet recruiting
Cetuximab + Adagrasib (KRYSTAL-10)	NCT04793958	3	Advanced CRC progressed on the first line of chemotherapy	Active, not recruiting
Cetuximab + JDQ443 (KontRASt-03)	NCT05358249	1/2	Advanced solid tumors	Recruiting
Cetuximab + JAB21822	NCT05194995	1/2	Advanced CRC, small intestine and appendiceal cancer	Recruiting
Cetuximab + IBI351	NCT05497336	1	Metastatic CRC	Recruiting
Cetuximab + Adagrasib + Irinotecan	NCT05722327	1	Advanced CRC	Recruiting
Erlotinib + Divarasib	NCT04449874	1	Advanced solid tumors	Recruiting

CRC: Colorectal Cancer

* Current status according to ClinicalTrials.gov as of 4/16/2024

## Abbreviations

CRC: Colorectal Cancer
FDA: Food and Drug Administration
KRAS: Kristen Rat Sarcoma Viral Oncogene Homologue
mPFS: Median Progression-Free Survival
ORR: Objective Response Rate
RTK: Receptor Tyrosine Kinase
